# Clinical phenotypes of depressed patients with evidence of inflammation and somatic symptoms

**DOI:** 10.1016/j.cpnec.2021.100079

**Published:** 2021-08-05

**Authors:** Éimear M. Foley, Joel T. Parkinson, Nils Kappelmann, Golam M. Khandaker

**Affiliations:** a Department of Psychiatry, University of Cambridge, Cambridge, UK; b Institute of Infection, Immunity and Inflammation, University of Glasgow, Glasgow, UK; c Department of Research in Translational Psychiatry, Max-Planck-Institute of Psychiatry, Munich, Germany; d International Max Planck Research School for Translational Psychiatry (IMPRS-TP), Munich, Germany; e MRC Integrative Epidemiology Unit, Population Health Sciences, Bristol Medical School, University of Bristol, Bristol, UK; f Centre for Academic Mental Health, Population Health Sciences, Bristol Medical School, University of Bristol, Bristol, UK; g Cambridgeshire and Peterborough NHS Foundation Trust, Cambridge, UK; h Avon and Wiltshire Mental Health Partnership NHS Trust, Bristol, UK

**Keywords:** C-Reactive protein, Inflammation, Depression, Fatigue, Anxiety, Quality of life

## Abstract

Whether depressed patients with evidence of inflammation are more appropriate candidates for immunotherapies is being tested in several clinical trials, which are selecting patients based on elevated C-reactive protein (CRP) and inflammation-related symptoms. However, studies of the clinical and phenotypic profile of depressed patients with elevated CRP are relatively scarce. We have investigated detailed clinical characteristics of 84 depressed patients, grouped as those with (CRP≥3 mg/L) and without (CRP<3 mg/L) inflammation. All patients met the International Classification of Diseases 10th Revision criteria for current depressive episode and had somatic symptoms of depression. We report that depressed patients with inflammation are more likely to be older (*P*=0.04), have higher body mass index (*P*<0.01), and be on non-selective serotonin reuptake inhibitor antidepressants (*P*=0.04). After adjusting for potential confounders, the inflammation group had higher depression severity (adjusted mean difference, 8.82; 95% CI, 3.91–13.72), somatic symptoms (adjusted mean difference, 3.25; 95% CI, 1.58–4.92), state anxiety (adjusted mean difference, 9.25; 95% CI, 3.82–14.67), perceived stress (adjusted mean difference, 4.58; 95% CI, 1.98–7.18), and fatigue (adjusted mean difference, 9.71; 95% CI, 3.09–6.33), but not anhedonia. The inflamed group also had poorer quality of life (adjusted mean difference, −0.18; 95% CI, −0.32–0.05). At individual depressive symptom level, the inflammation group had increased guilty feelings (adjusted odds ratio [OR], 7.28; 95% CI, 2.09–31.17), pessimism (adjusted OR, 5.38; 95% CI, 1.53–22.73), concentration difficulties (adjusted OR, 4.56; 95% CI, 1.32–19.02), and indecisiveness (adjusted OR, 4.21; 95% CI, 1.15–18.54). Our findings highlight the clinical features associated with inflammation in depressed patients with somatic symptoms, including poor quality of life, supporting the need for intervention targeting this group. These results could also aid patient and outcome selection in future clinical trials testing immunotherapies in depression. Replication of these findings in larger samples is required.

## Highlights

• We studied clinical features of depressed patients with somatic symptoms with/without evidence of inflammation .
• Elevated CRP (≥3mg/L) was associated with higher age, higher BMI, and non-SSRI treatment.
• Elevated CRP (≥3mg/L) was associated with higher depression severity, fatigue, state anxiety, and stress.
• Elevated CRP (≥3mg/L) was associated with poorer subjective wellbeing and quality of life.
• Elevated CRP (≥3mg/L) was associated with both somatic and psychological symptoms of depression.

## 1 Introduction

Accumulating evidence suggests an association between systemic low-grade inflammation and depression. Meta-analyses of cross-sectional studies confirm elevated concentrations of circulating inflammatory cytokines and acute phase proteins, like interleukin-6 (IL-6) and C-reactive protein (CRP), in patients with depression as compared to controls [19,49]. CRP is an archetypal inflammatory marker and has been used most extensively as a measure of inflammation in depression. A recent meta-analysis by Ref. [40] reported that evidence of low-grade inflammation (i.e., CRP >3 mg/L) is present in approximately 27% of depressed patients. Emerging evidence from population-based prospective studies and genetic Mendelian randomization (MR) studies suggest that inflammation may play a causal role in depression [29,30,55]. Inflammation is also clinically relevant and has been associated with poor response to antidepressants [8,36]. Additionally, anti-inflammatory drugs have antidepressant effects in patients with chronic inflammatory conditions, where novel anti-cytokine drugs improve depressive symptoms (at least partly) independently of improving physical illness symptoms [26,32,54]. These findings highlight the potential for therapeutic targeting of inflammation in patients with depression.

Currently, a number of randomised controlled trials (RCTs) are testing the effects of immunotherapies for depression outside the context of major physical illnesses. However, which depressed patients are likely to benefit from such treatment remains a key outstanding question. A RCT of infliximab [44], a tumour necrosis factor alpha (TNF-α) specific monoclonal antibody, reported that patients with evidence of inflammation may be more suitable candidates for immunotherapy trials. Consequently, a number of RCTs have begun evaluating immunotherapies on depressed patients with evidence of inflammation [2,28,51]. Typically, these trials have defined inflammation as elevated CRP or IL-6 levels. However, there is relatively limited research into potential characteristics of depressed patients with evidence of inflammation. Such characteristics could help inform patient selection in future RCTs.

Evidence suggests that elevated CRP levels in depressed patients are associated with higher depression severity [31] and somatic symptoms (e.g., fatigue, changes in appetite, and sleep), rather than psychological symptoms (e.g., hopelessness, excessive/inappropriate guilt) [24,39]. However, existing studies have often used single items on depression scales as measures of fatigue and anhedonia. In-depth assessment of these key inflammation-related symptom domains is required as fatigue and anhedonia are complex multi-faceted traits [12,53]. Anxiety symptoms are also often present in patients with depression, but studies testing the association between CRP levels and anxiety symptoms in patients with depression are scarce. Furthermore, little is known about associations of CRP levels with perceived stress, quality of life, and subjective wellbeing, which are key indicators of overall wellbeing.

To further understand the characteristics of depressed patients with somatic symptoms with and without evidence of inflammation, we have investigated a range of sociodemographic factors, clinical history, affective and somatic symptoms, quality of life, and wellbeing in these samples. We hypothesised that inflammation would be associated with a distinct phenotypic profile characterised by higher depression severity, increased fatigue, anhedonia, and poorer quality of life.

## 2 Materials and methods

### 2.1 Participants and study design

A case-control design was applied involving participants with International Classification of Diseases 10th Revision (ICD-10) diagnosis of depressive episode (ICD-10 code F32) and somatic symptoms (see below), grouped as with or without evidence of inflammation based on CRP levels. We aimed to recruit roughly equal number of participants with low and high CRP; recruitment strategy for both groups was identical. All patients were recruited through UK National Health Service Mental Health Trusts, primary care general practice surgeries, and self-referral in the East Anglia region between October 2018 and March 2020 as part of a RCT [28]. All participants were required to meet the following criteria regardless of their CRP levels: aged 20–65 years, ICD-10 criteria for depressive episode at time of assessment (confirmed by the Clinical Interview Schedule – Revised), somatic symptom score ≥7 (based on Beck Depression Inventory II, or BDI-II, items on pleasure, energy, sleep, appetite, concentration difficulty, tiredness/fatigue, and libido), currently taking an antidepressant at an adequate dose (as determined by British National Formulary or BNF) for at least four weeks. Exclusion criteria were current or lifetime diagnosis of bipolar disorder, psychotic disorder, personality disorder, eating disorder, history of alcohol or substance abuse/dependence within six months prior to assessment (nicotine and caffeine dependence were not exclusionary), current suicidal thoughts or wishes (assessed by BDI-II item 9 score of 3) or history of suicide attempt or deliberate self-harm within six months prior to assessment, any current infection, any infection requiring hospitalisation or treatment with intravenous antibiotics within four weeks prior to assessment, pregnancy or breast feeding, and physical illness and/or use of medication likely to compromise interpretation of immunological data. Self-reported information on key inclusion/exclusion criteria were verified by the participant's general practitioner prior to enrolment.

Demographic and medical history information was recorded and self-administered psychiatric evaluations were completed during the study visit. Additionally, all participants provided blood samples for serum high sensitivity CRP (hs-CRP) measurement. The sample was divided into two groups according to hs-CRP levels: those with evidence of low-grade inflammation (hs-CRP ≥3 mg/L), and those without evidence of low-grade inflammation (hs-CRP <3 mg/L). This cut-off was chosen based on the American Heart Association and Center for Disease Control and Prevention recommendations, which defined CRP levels of >3 mg/L as high [41].

All participants provided informed consent. The study was approved by the South Central – Oxford B Research Ethics Committee (Reference: 18/SC/0118).

### 2.2 Measurement of CRP

Blood samples were collected from non-fasting participants. Samples were promptly centrifuged and assayed for serum hs-CRP levels using an automated colorimetric immunoassay on the Siemens Dimension EXL analyser. The minimum detection limit was 0.1 mg/L. All samples were assayed at the Core Biochemical Assay Laboratory, located in Addenbrooke's Hospital, Cambridge, by staff blind to psychiatric measure outcomes. Acute infection was excluded by white blood cell count, antibody tests for TB, HIV, Hepatitis B, and Hepatitis C, and chest X-ray.

### 2.3 Diagnosis of depression

ICD-10 diagnosis of depression was assessed by the Clinical Interview Schedule – Revised (CIS-R), which was administered by trained research staff. The CIS-R is a widely used, standardised tool for measuring common mental health disorders in research settings [35]. The CIS-R is a fully structured assessment suitable for trained social survey interviewers and does not require any expert knowledge on the part of the interviewers. As such, it can also be administered using personal computers on which the subjects self-complete the questionnaire [35]. The CIS-R elicits responses to 14 areas of symptoms including fatigue, appetite, sleep problems, concentration difficulties, irritability, depression, depressive ideas, anxiety, worry, panic, phobia, compulsive behaviours, obsessive thoughts, and somatic symptoms. It can be used to generate diagnostic categories according to the ICD-10, including diagnosis of depression.

### 2.4 Assessment of outcome measures

Participants completed self-administered validated questionnaires for depression, state and trait anxiety, perceived stress, pleasure, fatigue, quality of life, and subjective wellbeing. Total scores for each of these were used as main outcomes (continuous variables), calculated by summing individual item scores according to user manuals. For all questionnaires higher scores represented greater symptom severity, with the exceptions of subjective wellbeing and quality of life (see below). We used Cronbach's alpha to quantify internal consistency in the current study [11]; Cronbach's alpha of ≥0.90 was considered as excellent, 0.8–0.9 as good, 0.7–0.8 as acceptable, 0.6–0.7 as questionable, 0.5–0.6 as poor, and <0.5 as unacceptable.

#### 2.4.1 Depressive symptoms

Depressive symptoms were assessed using the BDI-II [1]. Each item on this 21-item questionnaire was coded on a 4-point scale ranging from 0 to 3 giving a total score of 0–63. Cronbach's alpha for the BDI-II was 0.88, indicating high internal consistency in this sample. In addition to total score, we created a categorical variable representing different degrees of depression severity using established thresholds for the total score as follows: 0–13 = minimal/no depression, 14–19 = mild depression, 20–28 = moderate depression, and 29–63 = severe depression [1,50]. Using the BDI-II cut-off of 14, sensitivity and specificity for depression were reported as 87.7% and 83.9% respectively in adults in primary care [50]. A cut-off of 20 has similar sensitivity and specificity for depression, 82% and 75% respectively, as reported in a sample of adult outpatients [50].

Furthermore, we calculated a somatic symptom score by summing seven relevant BDI-II items based on current literature suggesting a link between inflammation and somatic symptoms of depression [9,24,39], specifically: 4 = lack of pleasure, 15 = loss of energy, 16 = changes in sleeping pattern, 18 = changes in appetite, 19 = concentration difficulty, 20 = tiredness or fatigue, and 21 = loss of interest in sex. Cronbach's alpha for the somatic symptom score was 0.69, indicating acceptable internal consistency in this sample. For symptom-based analyses, we recoded each symptom as a binary variable by recoding scores 0 and 1 to represent no/mild symptoms and scores 2 and 3 to represent moderate/severe symptoms. In depression, sleep and appetite changes can occur in either direction. Therefore, in addition to a composite variable reflecting change, we created separate variables to represent an increase or decrease in both sleep and appetite.

#### 2.4.2 Anxiety, stress, pleasure, and fatigue

The State-Trait Anxiety Inventory (STAI) [47] was used to measure state (STAI-S) and trait (STAI-T) anxiety. Both questionnaires demonstrated good internal reliability with a Cronbach's alpha of 0.92 and 0.88, respectively. Participants were presented with two 20-item questionnaires to assess these two forms of anxiety. Responses were recorded on a 4-point scale ranging from 1 to 4, giving a total score of 20–80 per questionnaire. Stress was assessed using the Perceived Stress Scale-10 (PSS) [10], a 10-item questionnaire where each item is coded as 0 to 4 with a total score of 0–40 (Cronbach's α = 0.83). The Snaith-Hamilton Pleasure Scale (SHAPS) [46] was used to measure anhedonia. Items on this 14-item scale were coded as 0 = agree and 1 = disagree with a total score of 0–14 (Cronbach's α = 0.82). Fatigue was assessed using the 20-item Multidimensional Fatigue Inventory (MFI) [45]. Item scores were coded on a 5-point scale ranging from 1 to 5 with a total score of 20–100 (Cronbach's α = 0.91). Higher scores indicated greater fatigue severity. The MFI evaluates five dimensions of fatigue, namely general fatigue (Cronbach's α = 0.71), physical fatigue (Cronbach's α = 0.84), mental fatigue (Cronbach's α = 0.84), reduced motivation (Cronbach's α = 0.63), and reduced activity (Cronbach's α = 0.86) with score ranges of 5–20. In addition to MFI total score, we calculated total scores for each of these five individual dimensions.

#### 2.4.3 Subjective wellbeing and quality of life

Subjective well-being was assessed using the Visual Analogue Scales for Subjective Well-Being (VAS-W) [4]. Each item in this series of 16 analogue scales was assigned a score between 1 and 100, representing a participant's response to the item in question (Cronbach's α = 0.90). The VAS-W consists of three dimensions of wellbeing; alertness (Cronbach's α = 0.83), contentedness (Cronbach's α = 0.84), and calmness (Cronbach's α = 0.52, reflecting poor internal consistency for this dimension). Total scores for each dimension were calculated by summing the representative item scores for each scale.

Quality of life was assessed using the EQ-5D three-level version (EQ-5D-3L) [15]. The EQ-5D-3L assesses five dimensions of quality of life, namely, mobility, self-care, usual activities, pain/discomfort, and anxiety/depression (Cronbach's α = 0.65). Participants assigned each dimension a score ranging from 1 to 3, with higher scores indicating poorer quality of life. Combining these numbers in sequence resulted in a five-digit health state profile that represented the level of reported problems on each of the five dimensions. Health state profiles were converted into a single index value to reflect participants' overall quality of life, scored from around 0 to maximum 1, with 1 representing perfect health.

### 2.5 Assessment of covariates

We used self-report questionnaires to measure age, sex, ethnicity, relationship status, employment status, alcohol, tobacco and other drug use, physical comorbidity, number of previous depressive episodes, current antidepressant type and treatment duration. Body mass index (BMI) was calculated from height and weight, which were assessed during the study visit. Regression models were adjusted for age (years), sex (male/female), BMI, and type of current antidepressant medication (selective serotonin reuptake inhibitors/SSRIs or other) as these variables were statistically different between the two groups. Other covariates, including alcohol and tobacco use frequency, were not significantly different between groups, and thus were not adjusted for in the statistical models.

### 2.6 Statistical analysis

Statistical analyses were performed in R version 4.0.2 [43]. The sample comprised 84 participants, grouped as 40 with elevated CRP (≥3 mg/L) and 44 with low CRP (<3 mg/L). This sample size allowed 80% statistical power to detect moderate-to-large effect sizes of Cohen's *d* = 0.62 (two-sided tests; alpha = 0.05). Statistical significance was defined by *P*<0.05, with false discovery rate (FDR)-adjusted corrections for multiple comparisons for each set of analyses using the Benjamini-Hochberg method [3].

#### 2.6.1 Sample characteristics

Sociodemographic and other characteristics were compared between two groups of patients with and without evidence of inflammation. Mean values for continuous variables were compared using independent samples *t*-test. For categorical variables, the proportion of participants between groups were compared using the Chi-squared test. Variables that violated the assumption of normality (i.e., BMI) were log-transformed before testing significance due to right skew.

#### 2.6.2 Association of inflammation with psychiatric measures and quality of life

Independent samples *t*-test and analysis of covariance (ANCOVA) were used to assess mean difference in scores for depression, other psychiatric, and quality of life measures between groups before and after adjusting for potential confounders, including age, sex, BMI, and type of current antidepressant medication. For categorical variables (e.g., depression category), proportion of participants between groups were compared using Chi-squared test. Variables that violated the assumption of normality (i.e., MFI total score, EQ-5D-3L index total score, and MFI general fatigue score) were square-transformed before testing significance due to right skew.

#### 2.6.3 Association between CRP and depressive symptoms

Logistic regression was used to estimate the odds ratio (OR) and 95% confidence interval (CI) for each BDI-II depressive symptom, coded as binary variables, for participants with evidence of inflammation compared to those without. Regression models were adjusted for age, sex, BMI, and current antidepressant medication type. Adjusted ORs (95% CIs) were visualised using forest plots.

## 3 Results

### 3.1 Sample characteristics

This study included 84 patients: 40 with evidence of low-grade inflammation (median hs-CRP = 7.32 mg/L; interquartile range = 4.45, 10.64) and 44 without (median hs-CRP = 0.66 mg/L; interquartile range = 0.38, 1.39). The group with evidence of inflammation was older (*P* = 0.04), had higher BMI (*P*<0.01), and was more likely to be on a non-SSRI antidepressant (*P*=0.04) (Table 1).

**Table 1:** Characteristics of patients with current ICD-10 depressive episode and somatic symptoms included in the study (N=84).

Characteristic	Evidence of inflammation		Test statistic (*P*-value^a^)
	Yes (hs-CRP ≥3mg/L)	No (hs-CRP <3mg/L)	
Sample, no. (%)	40 (48)	44 (52)	-
hs-CRP, mean (SD)	8.22 (4.95)	0.92 (0.75)	-
hs-CRP, median (IQR)	7.32 (4.45, 10.64)	0.66 (0.38, 1.39)	-
Age, mean (SD)	41.30 (11.16)	35.97 (12.08)	2.10 (0.04)
Female sex, no. (%)	28 (70)	32 (73)	0.08 (0.78)
BMI, mean (SD)^b^	35.93 (8.07)	25.62 (6.45)	7.21 (<0.01)^c^
Ethnicity, no. (%)			
White	37 (93)	41 (93)	0.02 (0.90)
Other	3 (7)	3 (7)	
Relationship status, no. (%)			
Relationship	24 (60)	25 (57)	0.09 (0.77)
Single	16 (40)	19 (43)	
Employment status, no. (%)			
Employed	32 (80)	31 (70)	1.02 (0.31)
Unemployed	8 (20)	13 (30)	
Previous depressive episodes, no. (%)			
2 or less	10 (25)	11 (25)	0.02 (1.00)
3 – 4	6 (15)	7 (16)	
5 or more	16 (40)	17 (39)	
Don’t know	8 (20)	9 (20)	
Current antidepressant duration, no. of months (SD)^b^	20.84 (37.19)	23.65 (38.26)	0.34 (0.74)
Total past antidepressant medication, no. (%)			
1	18 (45)	23 (52)	4.69 (0.10)
2	18 (45)	11 (25)	
3 or more	4 (10)	10 (23)	
Current antidepressant type, no. (%)			
SSRI	25 (62)	36 (82)	3.93 (0.04)
Other	15 (38)	8 (18)	
Alcohol use frequency, no. (%)			
Never	12 (30)	8 (18)	2.60 (0.27)
Monthly/Yearly	19 (48)	20 (46)	
More than once per week	9 (22)	16 (36)	
Tobacco use frequency, no. (%)			
Never	28 (70)	26 (59)	2.21 (0.33)
Less than daily	3 (8)	8 (18)	
Daily	9 (22)	10 (23)	
Other drug use, no. (%)	6 (15)	13 (30)	2.53 (0.11)
Physical comorbidity, no. (%)^d^			
0	9 (26)	18 (41)	2.31 (0.31)
1	13 (37)	15 (34)	
2 or more	13 (37)	11 (25)	

ICD-10, International Classification of Diseases 10th Revision; hs-CRP, high-sensitivity C-reactive protein; SD, standard deviation; IQR, interquartile range; BMI, body mass index; SSRI, selective serotonin reuptake inhibitors.

a Mean values for continuous variables were compared using independent samples *t*-test; For categorical variables proportion of participants between groups were compared using Chi-squared test.

b N=83

c Variables that violated the assumption of normality (i.e., BMI) were log-transformed before testing significance due to right skew.

d N=79

### 3.2 Association of inflammation with psychiatric measures

**Fig. 1:**
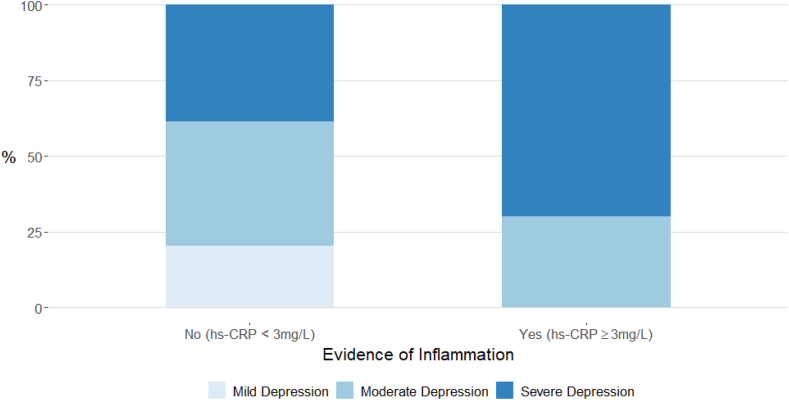
Cases of ICD-10 current mild, moderate, and severe depressive episode grouped by serum hs-CRP level. ICD-10, International Classification of Diseases 10th Revision; hs-CRP, high-sensitivity C-reactive protein.

The group with inflammation had higher BDI-II total scores (adjusted mean difference, 8.82; 95% CI, 3.91, 13.72; adjusted *P*=0.02), somatic symptom scores (adjusted mean difference, 3.25; 95% CI, 1.58, 4.92; adjusted *P*=0.02), state anxiety (adjusted mean difference, 9.25; 95% CI, 3.82, 14.67; adjusted *P*=0.02), perceived stress (adjusted mean difference, 4.58; 95% CI, 1.98, 7.18; adjusted *P*=0.02) (Table 2), and increased depression severity (χ^2^ = 7.78; adjusted *P*=0.03) (Fig. 1). The group with inflammation also had increased scores for total fatigue (adjusted mean difference, 9.71; 95 % CI, 3.09, 6.33; adjusted *P*=0.02), general fatigue (adjusted mean difference, 1.82; 95 % CI, 0.51, 3.13; adjusted *P*=0.03), and physical fatigue (adjusted mean difference, 3.99; 95 % CI, 1.99, 5.98; adjusted *P*=0.03) (Table 3). The inflamed group also had significantly higher scores for mental fatigue (unadjusted mean difference, 1.73; 95 % CI, 0.32, 3.13), reduced motivation (unadjusted mean difference, 1.76; 95 % CI, 0.51, 3.01), and reduced activity (unadjusted mean difference, 2.61; 95 % CI, 0.82, 4.40) in the unadjusted analysis.

**Table 2:** Affective symptoms, subjective wellbeing, and quality of life measures in depressed patients with somatic symptoms, grouped as with or without inflammation based on CRP.

Measures	Evidence of inflammation		Mean Difference (95% CI)^a^			T-value^b^ (*P*-value) for final model	Corrected^c^*P*-value for final model
	Yes (n = 40; hs-CRP ≥3 mg/L) Mean (SD)	No (n = 44; hs-CRP <3 mg/L) Mean (SD)	Unadjusted	Adjusted for age, sex, BMI	Additional adjustment for antidepressant type		
BDI-II total score	34.63 (9.02)	27.55 (9.19)	7.08 (3.12, 11.04)	9.25 (4.22, 14.29)	**8.82 (3.91, 13.72)**	3.58 (<0.01)	0.02
BDI-II somatic symptom score	12.23 (2.96)	9.16 (3.26)	3.07 (1.71, 4.42)	3.40 (1.69, 5.11)	**3.25 (1.58, 4.92)**	3.88 (<0.01)	0.02
Trait anxiety (STAI-T) total score	62.50 (7.73)	60.11 (9.21)	2.39 (−1.33, 6.10)	4.21 (−0.51, 8.94)	4.05 (−0.69, 8.80)	1.70 (0.09)	0.11
State anxiety (STAI-S) total score	60.00 (10.12)	53.27 (9.58)	6.73 (2.45, 11.00)	9.45 (4.04, 14.86)	**9.25 (3.82, 14.67)**	3.39 (<0.01)	0.02
PSS total score	28.53 (4.65)	25.18 (5.26)	3.34 (1.18, 5.51)	4.82 (2.15, 7.48)	**4.58 (1.98, 7.18)**	3.51 (<0.01)	0.02
SHAPS total score	5.35 (3.49)	4.14 (3.46)	1.21 (−0.30, 2.72)	1.02 (−0.88, 2.92)	0.87 (−1.00, 2.73)	−0.93 (0.36)	0.36
MFI total score	82.75 (9.52)	70.45 (13.96)	12.30 (7.14, 17.45)	10.17 (3.47, 16.86)	**9.71 (3.09, 16.33)**	2.92 (<0.01)^d^	0.02
EQ-5D-3L total index score	0.49 (0.29)	0.69 (0.22)	−0.20 (−0.32, −0.09)	−0.20 (−0.34, −0.60)	**−0.18 (-0.32, -0.05)**	2.89 (<0.01)^d^	0.02
VAS-W alertness total score	61.64 (10.58)	57.05 (10.66)	4.59 (−0.03, 9.21)	5.43 (−0.54, 11.39)	4.90 (−0.91, 10.70)	0.68 (0.10)	0.11
VAS-W contentedness total score	62.54 (13.20)	56.93 (12.12)	5.61 (0.11, 11.10)	7.03 (0.01, 14.05)	6.43 (−0.43, 13.28)	1.87 (0.07)	0.09
VAS-W calmness total score	33.78 (8.52)	41.45 (6.75)	−7.68 (−11.00, −4.36)	−8.64 (−12.88, −4.40)	**−8.48 (-12.73, -4.22)**	3.97 (<0.01)	0.02

hs-CRP, high-sensitivity C-reactive protein; SD, standard deviation; BMI, body mass index; BDI-II, Beck Depression Inventory II; STAI-T, State-Trait Anxiety Inventory – Trait; STAI-S, State-Trait Anxiety Inventory – State; PSS, Perceived Stress Scale-10; SHAPS, Snaith-Hamilton Pleasure Scale; MFI, Multidimensional Fatigue Inventory; EQ-5D-3L, EQ-5D three-level version; VAS-W, Visual Analogue Scales for Subjective Well-Being.

a Total sample for adjusted models is N = 83.

b Mean values for continuous variables were compared using independent samples *t*-test and ANCOVA.

c P-values corrected for multiple testing using Benjamini & Hochberg’s False Discovery Rate method.

d Variables that violated the assumption of normality (i.e., MFI total score and EQ-5D-3L index total score) were square-transformed before testing significance due to left skew.

**Table 3:** Fatigue dimension scores in depressed patients with somatic symptoms, grouped as with or without inflammation based on CRP.

MFI Fatigue Dimensions	Evidence of inflammation		Mean Difference (95% CI)^a^			Test Statistic^b^ (*P*-value) for final model	Corrected^c^*P*-value for final model
	Yes (n = 40; Mean (SD)	No (n = 44; Mean (SD)	Unadjusted	Adjusted for age, sex, BMI	Additional adjustment for antidepressant type		
General fatigue	18.40 (1.43)	16.55 (2.94)	1.86 (0.84, 2.87)	1.88 (0.57, 3.19)	**1.82 (0.51, 3.13)**	2.85 (<0.01)^d^	0.03
Physical fatigue	16.80 (2.78)	12.45 (4.20)	4.35 (2.78, 5.91)	4.12 (2.10, 6.13)	**3.99 (1.99, 5.98)**	3.98 (<0.01)	0.03
Mental fatigue	15.60 (2.54)	13.84 (3.16)	1.73 (0.32, 3.13)	1.44 (−0.34, 3.23)	1.35 (−0.43, 3.13)	1.51 (0.14)	0.20
Reduced motivation	15.23 (3.54)	12.61 (4.59)	1.76 (0.51, 3.01)	1.00 (−0.60, 2.59)	0.90 (−0.68, 2.49)	1.14 (0.26)	0.26
Reduced activity	16.73 (2.78)	15.00 (3.58)	2.61 (0.82, 4.40)	1.73 (−0.56, 4.02)	1.65 (−0.64, 3.95)	1.43 (0.16)	0.20

MFI, Multidimensional Fatigue Inventory; hs-CRP, high-sensitivity C-reactive protein; SD, standard deviation; BMI, body mass index.

a Total sample for adjusted models was N = 83.

b Mean values for continuous variables were compared using independent samples *t*-test and ANCOVA.

c P-values corrected for multiple testing using Benjamini & Hochberg’s False Discovery Rate method.

d Variables that violated the assumption of normality (i.e., MFI general fatigue) were square-transformed before testing significance due to left skew.

### 3.3 Association of inflammation with subjective wellbeing and quality of life

The group with inflammation had lower scores for calmness on the VAS-W (adjusted mean difference, −8.48; 95 % CI, −12.73, −4.22; adjusted *P*=0.02), and poorer quality of life (adjusted mean difference, −0.18; 95 % CI, −0.32, −0.05; adjusted *P*=0.02) (Table 2).

### 3.4 Association between CRP and individual depressive symptoms

**Fig. 2:**
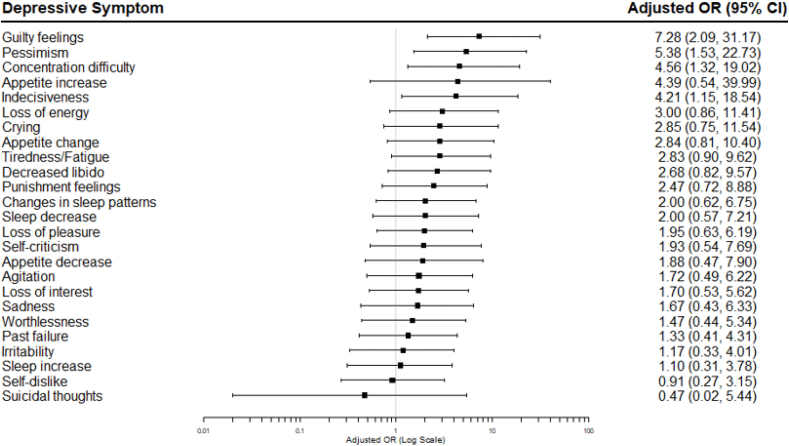
Adjusted (i.e., age, sex, BMI, and current antidepressant medication type) Odds Ratios (95% CI) for individual depressive symptoms in depressed patients with somatic symptoms and evidence of inflammation. OR, Odds ratio.

At symptom level, the group with inflammation had higher odds for guilty feelings (adjusted OR, 7.28; 95% CI, 2.09, 31.17; unadjusted *P*<0.01), pessimism (adjusted OR, 5.38; 95% CI, 1.53, 22.73; unadjusted *P*=0.01), concentration difficulty (adjusted OR, 4.56; 95% CI, 1.32, 19.02; unadjusted *P*=0.02), and indecisiveness (adjusted OR, 4.21; 95% CI, 1.15, 18.54; unadjusted *P*=0.04) (Table 4; Fig. 2). However, these no longer remained significant following FDR correction. The group with inflammation also had higher odds for loss of energy (unadjusted OR, 3.00; 95% CI, 1.21,7.83), appetite change (unadjusted OR, 4.07; 95% CI, 1.56, 11.42), tiredness/fatigue (unadjusted OR, 2.92; 95% CI, 1.22, 7.23), punishment feelings (unadjusted OR, 3.52; 95% CI, 1.45, 9.19), loss of pleasure (unadjusted OR, 2.95; 95% CI, 1.23, 7.33), and appetite decrease (unadjusted OR, 4.20; 95% CI, 1.42, 14.30) in the unadjusted analysis, before FDR correction. It is worth noting that after adjusting for potential confounders, while the point estimates remained similar, confidence intervals widened and included the null. The inflammation group also showed higher odds for appetite increase (adjusted OR, 4.21; CI, 0.54, 39.99), but sample sizes for this symptom were low and the difference between groups was not significant.

**Table 4:** Odds Ratios (95% CI) for individual depressive symptoms in depressed patients with somatic symptoms and evidence of inflammation.

Depressive Symptom	Evidence of inflammation		Odds Ratio (95% CI) for Depressive Symptom^a^			Test Statistic (*P*-value)	Corrected^b^*P*-value for final model
	Yes (hs-CRP ≥3 mg/L) N (%) with symptom	No (hs-CRP <3 mg/L) N (%) with symptom	Unadjusted	Adjusted for age, sex, BMI	Additional adjustment for antidepressant type		
Guilty feelings	28 (70)	6 (14)	4.08 (1.67, 10.48)	7.33 (2.11, 31.31)	**7.28 (2.09, 31.17)**	**2.93 (**< **0.01)**	0.13
Pessimism	23 (58)	15 (34)	2.62 (1.09, 6.45)	5.25 (1.54, 21.09)	**5.38 (1.53, 22.73)**	**2.48 (0.01)**	0.13
Concentration difficulty	28 (70)	22 (50)	2.33 (0.96, 5.86)	4.61 (1.34, 19.19)	**4.56 (1.32, 19.02)**	**2.27 (0.02)**	0.17
Appetite increase	5 (13)	3 (7)	1.95 (0.45, 10.07)	4.38 (0.59, 36.21)	4.39 (0.54, 39.99)	1.36 (0.17)	0.39
Indecisiveness	30 (75)	26 (59)	2.08 (0.83, 5.44)	4.20 (1.18, 17.92)	**4.21 (1.15, 18.54)**	**2.06 (0.04)**	0.25
Loss of energy	30 (75)	22 (50)	3.00 (1.21, 7.83)	3.03 (0.89, 11.32)	3.00 (0.86, 11.41)	1.69 (0.09)	0.34
Crying	15 (38)	11 (25)	1.80 (0.71, 4.68)	2.73 (0.79, 10.23)	2.85 (0.75, 11.54)	1.53 (0.13)	0.36
Appetite change	19 (48)	8 (18)	4.07 (1.56, 11.42)	2.88 (0.84, 10.31)	2.84 (0.81, 10.40)	1.62 (0.10)	0.34
Tiredness/Fatigue	25 (63)	16 (36)	2.92 (1.22, 7.23)	2.86 (0.92, 9.47)	2.83 (0.90, 9.62)	1.74 (0.08)	0.34
Decreased libido	23 (58)	19 (43)	1.78 (0.75, 4.28)	2.71 (0.83, 9.64)	2.68 (0.82, 9.57)	1.59 (0.11)	0.34
Punishment feelings	19 (48)	9 (20)	3.52 (1.38, 9.54)	2.45 (0.72, 8.80)	2.47 (0.72, 8.88)	1.43 (0.15)	0.38
Changes in sleep patterns	25 (63)	22 (50)	1.67 (0.70, 4.04)	2.04 (0.65, 6.75)	2.00 (0.62, 6.75)	1.15 (0.25)	0.48
Sleep decrease	13 (33)	10 (23)	1.64 (0.63, 4.39)	2.03 (0.58, 7.28)	2.00 (0.57, 7.21)	1.09 (0.28)	0.50
Loss of pleasure	26 (65)	17 (39)	2.95 (1.23, 7.33)	1.98 (0.64, 6.27)	1.95 (0.63, 6.19)	1.15 (0.25)	0.48
Self-criticism	28 (48)	31 (53)	0.98 (0.38, 2.52)	1.96 (0.55, 7.81)	1.93 (0.54, 7.69)	0.99 (0.32)	0.53
Appetite decrease	14 (35)	5 (11)	4.20 (1.42, 14.30)	1.87 (0.46, 7.84)	1.88 (0.47, 7.90)	0.89 (0.37)	0.54
Agitation	11 (28)	12 (27)	1.01 (0.38, 2.65)	1.77 (0.52, 6.26)	1.72 (0.49, 6.22)	0.85 (0.40)	0.56
Loss of interest	20 (50)	17 (39)	1.59 (0.67, 3.82)	1.75 (0.57, 5.59)	1.70 (0.53, 5.62)	0.90 (0.37)	0.54
Sadness	13 (33)	8 (18)	2.17 (0.80, 6.18)	1.72 (0.47, 6.39)	1.67 (0.43, 6.33)	0.76 (0.45)	0.59
Worthlessness	25 (63)	28 (64)	0.95 (0.39, 2.32)	1.49 (0.45, 5.41)	1.47 (0.44, 5.34)	0.61 (0.54)	0.68
Past failure	26 (65)	23 (52)	1.70 (0.71, 4.14)	1.37 (0.43, 4.39)	1.33 (0.41, 4.31)	0.48 (0.63)	0.72
Irritability	17 (43)	15 (34)	1.43 (0.59, 3.49)	1.23 (0.37, 4.06)	1.17 (0.33, 4.01)	0.25 (0.81)	0.88
Sleep increase	12 (30)	12 (27)	1.14 (0.44, 2.97)	1.14 (0.33, 3.85)	1.10 (0.31, 3.78)	0.15 (0.88)	0.89
Self-dislike	26 (65)	28 (64)	1.06 (0.43, 2.62)	0.98 (0.30, 3.24)	0.91 (0.27, 3.15)	−0.14 (0.89)	0.89
Suicidal thoughts	2 (5)	3 (7)	0.72 (0.09, 4.57)	0.53 (0.03, 5.74)	0.47 (0.02, 5.44)	−0.57 (0.57)	0.68

hs-CRP, high-sensitivity C-reactive protein; BMI, body mass index.

a Total sample for adjusted models was N = 83.

b *P*-values corrected for multiple testing using Benjamini & Hochberg's False Discovery Rate method.

## 4 Discussion

We report the characteristics of depressed patients with somatic symptoms and evidence of inflammation, including an in-depth investigation into affective symptoms, fatigue, perceived stress, quality of life, and wellbeing. We replicated previous reports of inflammation being associated with higher depression severity. At symptom level, inflammation was found to be associated with both psychological and somatic symptoms of depression. Our work highlights particular domains of fatigue as being more strongly associated with inflammation, namely general and physical fatigue. We also show that depressed patients with somatic symptoms and evidence of inflammation have increased stress, lower subjective wellbeing, and poorer quality of life. These are clinically relevant findings that highlight the need for interventions targeting groups with inflammation-related depression.

Inflammation has previously been reported to be associated with various clinical features in patients with depression. Several studies have supported associations of higher CRP or IL-6 levels with increased depression severity [20,48]. A study of 231 depressed patients reported that prevalence of moderate/severe depressive episode was higher in those with elevated CRP (7–10 mg/L), as compared to those with low CRP (<1 mg/L) [31]. Our results for higher BDI-II total score and higher prevalence of categorically defined ICD-10 moderate/severe depression in the group with evidence of inflammation are consistent with these studies.

Using the BDI-II, we calculated a somatic symptom score comprising fatigue, loss of energy, anhedonia, changes in sleep or appetite, concentration difficulty, and decreased libido. This somatic symptom score was found to be higher in the group with inflammation-related depression, even though entry criteria required somatic symptoms to be present in all participants. Immune activation in cancer patients after treatment with interferon-alpha (IFN-α) has been shown to be associated with rapid development of somatic symptoms (namely fatigue and impaired sleep) in most patients, while other affective symptoms and cognitive dysfunction arise more slowly and in fewer patients [6,37]. Similarly, population-based studies have reported that elevated IL-6 and CRP levels are associated with fatigue and sleep disturbance, but not with psychological symptoms, such as hopelessness [24,38]. Similar findings have also been reported from the NESDA cohort, where inflammatory markers were found to be specifically associated with somatic symptoms of depression, such as fatigue, weight gain, and sleep disturbance [13]. However, in our symptom-level analysis, we found that elevated CRP was associated with both somatic (e.g., fatigue, loss of energy, concentration difficulties) and psychological symptoms (e.g., guilty feelings, pessimism, indecisiveness), before FDR correction. This could be due to overall higher depression severity in our sample, which comprised individuals meeting ICD-10 criteria for current depressive episode. It is possible that during early stages of depression inflammation is more relevant for somatic symptoms, while in established/chronic illness it is associated with both somatic and psychological symptoms of the syndrome. Of note, the current study excluded participants with severe suicidal thoughts or wishes (i.e., BDI II item 9 score = 3, “I would kill myself if I had the chance”). This may have led to lower severity of suicidal thoughts in our sample and introduced a bias towards the null for the results for suicidality in our symptom-level analysis.

While an association between inflammation and fatigue is well established [12,33], previous studies have typically used single items on depression scales rather than providing an in-depth assessment of specific dimensions of fatigue. We used the multidimensional fatigue inventory to examine specific fatigue domains. Our findings highlight that elevated CRP is associated with overall higher fatigue scores in depressed individuals, the domains of general and physical fatigue being most strongly associated with inflammation. Mental fatigue (i.e., cognitive symptoms of fatigue) was significantly higher in the inflamed group in the unadjusted model. A recent study reported that higher TNF-α levels were associated with cognitive fatigue, but not somatic or psychosocial fatigue in depressed individuals [42]. Taken together, these results suggest that inflammation may exacerbate not only a general feeling of fatigue, but also, specifically, physical and possibly cognitive fatigue in depressed individuals with somatic symptoms.

Anxiety symptoms are common in depression [23,27], yet few studies have examined the association of inflammation with anxiety in people with depression. We assessed state and trait anxiety, showing that elevated CRP is more strongly associated with state anxiety in depressed individuals. Of note, we excluded individuals with a primary diagnosis of anxiety disorder and any anxiety symptoms reported were in addition to the participants’ primary diagnosis of depression. These results support findings of an anxious-depression phenotype reported at transition to depression in a large cohort of IFN-α-treated patients [52]. More recently, higher concentrations of CRP were also found to be associated with increased prevalence of anxiety symptoms [55]. However, this association was fully attenuated after adjusting for depressive symptoms. Taken together, these results suggest that CRP is associated with anxiety, but these associations are strongly related to the presence of underlying depression. The results for increased state anxiety in our sample are consistent with that for stress, subjective wellbeing, and quality of life measures. We report that depressed individuals with evidence of inflammation had higher perceived stress, lower calmness scores, and poorer quality of life. A previous study also reported that physical quality of life was lower in depressed patients with chronic inflammation [16]. Our findings, together with existing evidence, suggest that inflammation may negatively impact the overall standard of health, comfort, and happiness experienced by depressed individuals with somatic symptoms and highlight the need for intervention.

In our sample of depressed individuals with somatic symptoms, elevated CRP was not associated with anhedonia as measured by the Snaith Hamilton Pleasure Scale. These findings are at odds with previous experimental studies reporting an association between inflammation and anhedonia/pleasure perception. In non-human primates, chronic low-dose infusion of IFN-α has been reported to decrease striatal dopamine release and increase anhedonia-like behaviour [18]. In patients with depression, plasma CRP concentration was reported to be associated with left basal ganglia glutamate levels, which, in turn, was associated with psychomotor slowing and anhedonia [17,21]. In healthy volunteers, experimental immuno-activation was reported to alter activation of reward-related brain regions [5,22], including reduction in the ventral striatum responses to hedonic reward [7,14]. Therefore, replication of our findings in larger samples is required.

Strengths of this work include in-depth assessments for affective symptoms, including depression, anxiety, anhedonia, and fatigue, as well as measures for stress, subjective wellbeing, and quality of life. We adjusted regression models for several relevant confounders including age, sex, BMI, and current antidepressant type. The primary limitation of this study is the relatively small sample size, limiting statistical power. This reduced the power of identifying small effect sizes, particularly after correcting for confounding and multiple comparisons. Nevertheless, this deep phenotyping study provides a useful starting point for the characterisation of depressed patients with evidence of inflammation and somatic symptoms. Second, over 70% of our sample was female and over 90% was White, reducing potential generalisability of our findings. Moreover, only depressed patients with somatic symptoms were recruited and so this sample is not representative of all cases of depression. Fourth, lack of a control group means that we cannot compare our results with non-depressed individuals. However, it is already known that affective symptoms and stress are higher and subjective wellbeing and quality of life is lower in depressed patients [25,34]. This study adds to current evidence by showing that some of these features are particularly relevant to depressed patients with evidence of inflammation and somatic symptoms.

## 5 Conclusion

In conclusion, our findings highlight that depressed individuals with somatic symptoms and evidence of low-grade systemic inflammation may have a distinct clinical profile, which includes higher depression severity, physical fatigue, state anxiety, and stress levels, as well as poorer quality of life and subjective wellbeing. These results add to our understanding of the phenotypic profile of inflammation-related depression and could help inform selection of patients and selection of key outcome measures in future RCTs of immunotherapies for depression. Replication of our findings in larger and more diverse samples is required.

## Declaration of competing interest

The authors declare that they have no known competing financial interests or personal relationships that could have appeared to influence the work reported in this paper.

## Role of the funding source

This work was funded by a Wellcome Trust fellowship to GMK (grant code: 201486/Z/16/Z). GMK also acknowledges funding support from Cambridgeshire and Peterborough NHS Foundation Trust R&D Department (Grant code: G101481), the BMA Foundation (J Moulton grant 2019); the MQ: Transforming Mental Health (grant code: MQDS17/40); and the Medical Research Council UK (grant codes: MC_PC_17,213 and MR/S037675/1). The BMA Foundation J Moulton grant supports ÉMF and the MRC grant MC_PC_17,213 supports JTP. NK is supported by the International Max Planck Research School of Translational Psychiatry (IMPRS-TP). The funding sources had no role in study design; collection, analysis, and interpretation of data; writing of the report; and the decision to submit the paper for publication.

## CRediT authorship contribution statement

**Éimear M. Foley:** Methodology, Formal analysis, Investigation, Data curation, Writing – original draft, Visualization, Project administration. **Joel T. Parkinson:** Formal analysis, Investigation, Writing – review & editing. **Nils Kappelmann:** Formal analysis, Writing – review & editing. **Golam M. Khandaker:** Conceptualization, Methodology, Formal analysis, Investigation, Resources, Writing – review & editing, Supervision, Project administration, Funding acquisition.
